# Effects of Dietary Phenylalanine and Tyrosine Supplements on the Chronic Stress Response in the Seabream (*Sparus aurata*)

**DOI:** 10.3389/fphys.2021.775771

**Published:** 2022-02-09

**Authors:** Natalia Salamanca, Oscar Moreno, Inmaculada Giráldez, Emilio Morales, Ignacio de la Rosa, Marcelino Herrera

**Affiliations:** ^1^IFAPA Centro Agua del Pino, Cartaya, Spain; ^2^Faculty of Experimental Sciences, University of Huelva, Huelva, Spain; ^3^Escuela Superior de Ingeniería, University of Huelva, Huelva, Spain

**Keywords:** *Sparus aurata*, stress, welfare, phenylalanine, tyrosine, feed supplement

## Abstract

The increase of aquaculture production is associated with a growing interest in improving physiological status and welfare in fish. For this reason, the search for strategies for mitigating stress has been intensified, with one of these strategies being food supplementation with different amino acids (AA). The objective of this study was to evaluate the effects of dietary phenylalanine (Phe) and tyrosine (Tyr) supplements on the endocrine and physiological state of seabreams (*Sparus aurata*) subjected to chronic stress. The fish were stocked at 30 fish/tank in a recirculation aquatic system, fed one control diet and two diets supplemented with 5% Phe or Tyr for 90 days. Blood was drawn from 10 fish per tank every 30 days, and the weight and length were measured every 15 days. At the end of the experiment, length/weight of the fish were measured, and they were sacrificed for the extraction of blood, head kidney, liver, and brain. Classic plasma stress markers (glucose, lactate, proteins, and cortisol), as well as hormones derived from Phe and Tyr (adrenaline, norepinephrine, and dopamine) and the accumulation of AA were analyzed. Fish fed with diets supplemented with Phe or Tyr showed a reduction in various stress markers and physiological parameters. In addition, the stress condition favored a mobilization of AA toward the tissues, especially in supplemented diets, so this excess of AA could be used as an energy substrate to cope with stress.

## Introduction

In recent years, aquaculture studies have focused on finding new strategies for minimizing stress and increasing animal welfare in the main species of interest, such as the gilthead seabream (*Sparus aurata*), whose culture is carried out in different marine farms along the Mediterranean and South-Atlantic areas, both onshore and offshore (AGAPA, 2019). In 2020, the production of seabream in the world experienced a decrease of 1.3% compared to 2020, reaching 249,200 tons, according to statistics from APROMAR (2020) (Spanish Association of Aquaculturists). Seabream farms exist in 18 countries, the top producers being Turkey (33.7% of total production), Greece (25.9% of total production), and Spain (9.3% of total production). Despite the position occupied by the Spanish production of seabream, it dropped off 9.4% in 2019, which represented an important decrease after the slight rebound occurred in 2017. The main Spanish region in seabream production is Valencia, which produces 49% of the total. Andalusia ranks third with a production of 1,606 tons in 2019. Besides important growth and reproductive indexes, this species’ success relies on its high domestication level (Teletchea, 2015).

Stress is a factor that may determine the success of fish culture. The primary physiological response to stress involves rises in plasma catecholamines and cortisol (Barton, 2002). Following, these hormones induce secondary responses, which are detected by the reduction in hepatic reserves (mainly glycogen) and increased plasma glucose levels, mobilizing glucose (and other energy substrates) to tissues for recovering homeostasis (Barton et al., 2002). If stress condition continues, the tertiary response can appear, being related to failures at organism level (reproduction, growth, survival, etc.) (Iwama et al., 2006).

Changes in salinity and environmental temperature, transport, and culture stocking density are some of the factors that can cause stress situations in marine farms, the most common being transport stress (acute stress) and culture stocking density (chronic stress) (Arends et al., 1999; Montero et al., 1999; Feidantsis et al., 2009; Martos-Sitcha et al., 2014; Jerez-Cepa et al., 2019). For this reason, stress reduction in fish has been widely studied using anesthetics, essential oils, or food additives (Ross and Ross, 2008; Zahl et al., 2012; Vanderzwalmen et al., 2018; Hoseini et al., 2019; Herrera et al., 2020; Salamanca et al., 2020, 2021).

Proteins constitute the essential part of the diet since they provide the amino acids (AA) necessary for the sustenance of the body. The AA which are a part of the cellular processes are called functional AA. A deficit of these AA can affect the body’s metabolism and homeostasis. In this sense, it has been shown that AA improve resistance to disease, reproduction, or behavior (Li et al., 2009; Wu, 2009). With the aim of optimizing the performance of aquaculture facilities and the quality of the final product, there has been an increase in the study of fish functional foods based on AA during the last years (Herrera et al., 2012, 2019; Andersen et al., 2016; Salamanca et al., 2020, 2021). In this sense, according to various authors, some amino acid dietary supplements could attenuate the stress response in different fish species and enhance the immune response (Herrera et al., 2016; Gonzalez-Silvera et al., 2018; Fernández-Alacid et al., 2019; Salamanca et al., 2020).

The effects of phenylalanine (Phe) and tyrosine (Tyr) have hardly been studied in fish (Herrera et al., 2016; Salamanca et al., 2021). Phe is an essential amino acid that is metabolized through two metabolic pathways, namely oxidation to Tyr and transamination to phenylpyruvate (Shafik et al., 2014). Tyr is a precursor of the catecholamine (adrenaline, noradrenaline, and dopamine) and thyroid (triiodothyronine and thyroxine) hormones, which are involved in the stress response (Reid et al., 1998). In this sense, it has been described that thermal stress rises the circulating levels of catecholamines in the rainbow trout (*Oncorhynchus mykiss*) (LeBlanc et al., 2011, 2012), and the concentration of plasma thyroid hormones could be a stress indicator (Hoseini et al., 2014). As stress is an energy-demanding process, Tyr also could be catabolized to hydroxyphenyl pyruvate and becomes a part of the energy metabolism.

It has been reported that Phe- and Tyr-enriched diets can mitigate the effects of acute stress in mammals (Brady et al., 1980; Lehnert et al., 1984; Banderet and Lieberman, 1989). In fact, Cotoia et al. (2014) have shown that hydroxyphenyl pyruvate improves cell survival under stress conditions in rats. In fish, both AA decrease the cases of bone deformity in *Diplodus sargus* larvae (Saavedra et al., 2010). Only Salamanca et al. (2021) have described the stress-attenuating effects of Phe and Tyr in meagre (*Argyrosomus regius*) and seabream (*S. aurata*) after submitting to acute stress. However, there are no studies on how Phe or Tyr supplements influence physiological responses to chronic stress. Therefore, the objective of this study is to analyze the effects of diets supplemented with Phe and Tyr on stress markers in situations of chronic stress in gilthead seabream (*S. aurata*).

## Materials and Methods

### Experimental Culture and Sampling

Gilthead seabreams *S. aurata* (*n* = 180), with a body weight of 33.93 ± 0.93 g and a total length of 12.87 ± 0.16 cm (mean ± standard error, SE), came from CULMASUR (Isla Cristina, Spain). The fish batch was divided into 30 fish/tank in six 500-L flat bottom circular tanks at a stocking density of 2 kg m^–3^. The culture water was recirculated, the flow rate, mean temperature, salinity, and dissolved oxygen levels were 300 L h^–1^, 21.47 ± 1°C, 37 ± 1 g L^–1^, and above 5 ppm, respectively. Water quality was checked weekly through Spectroquant kits (MERCK, Darmstadt, Germany), maintaining the following values (mg L^–1^) in each experimental tank: total ammonia <0.04; total nitrite <0.02; and total nitrate <0.03.

Before the experiment began, the fish were fed commercial feed (L2 Alterna^®^ Skretting, Burgos, Spain) with daily rations of 2% of biomass for 2 weeks. The experimental treatment consisted of two physiological statuses (basal and stress) fed with three types of feeding (control, food enriched with 5% Phe, and food enriched with 5% Tyr) for 90 days. The stressed groups were submitted to confinement and netting/chasing stress. This procedure consisted of fish that were randomly net-chased and netted (with no exposure to air) daily for 5 min three times a day (adapted from Gonzalez-Silvera et al., 2018 and Herrera et al., 2020). Those tanks had a water column of 20 cm, and thus an initial stocking density of 4 Kg m^–3^. The basal group tanks had a water volume of 500 L (40 cm water height, 2 kg m^–3^) and were not disturbed along the experiment. All tanks were supervised daily for cleaning (light siphoning). Fifteen fish from each tank were individually ink-tagged according to Rodiles et al. (2015) so the experimental unit was every individual fish (15 replicates) and tank replicates were not necessary.

Fish feed was provided through automatic feeders and the ration was adjusted to 2% of the tank biomass daily; the feeders supplied food 24 h a day. The rations were adjusted after each biometric sampling. The tanks were cleaned and checked daily for possible dead fish. Biometric samplings were carried out every 15 days and blood (0.1 mL) was drawn every 30 days, previously anesthetizing marked fish (*n* = 10) in a 2-phenoxyethanol bath (200 μL L^–1^). At the end of the 90-day trial, fish were sacrificed with 2-phenoxyethanol overdose (1 mL L^–1^) for blood and tissue extraction (10 fish per treatment). Prior to each sampling, the fish were fasted for 24 h and without manipulation that caused stress effects. Blood was collected from the caudal vein with 1 mL heparinized syringes (25,000 units of ammonium heparin/3 mL of 0.6% NaCl saline, Sigma H6279, Saint Louis, MO, United States). Plasma was separated from cells by whole blood centrifugation with an Eppendorf 5415R centrifuge (Hamburg, Germany) (3 min; 10,000 × *g*; 4°C) and stored at −80°C until analyzes were performed. The next biometric parameters were calculated: specific growth rate (% day^–1^), SGR = 100⋅(ln Wf − ln Wi)/t; conversion factor, CF = FS/(Wf − Wi); condition factor, *K* = 100⋅BW/TL3; and hepatosomatic index, HSI = 100⋅(Lw/BW); where Wf, Wi are individual final and initial weight (g); Lw is individual liver weight (g); BW and TL, individual body weight (g) and total length (cm); t, time (days); and FS is individual mean supplied food (g). No fish mortality was detected during the experiment.

The IFAPA facilities are certified and have the necessary authorization for the breeding and husbandry of animals for scientific purposes (REGA Code ES210210000303). All procedures involving the handling and treatment of the fish were approved as far as the care and use of experimental animals are concerned (authorization code 11/05/2021/073), by the European Union (2010/63/EU) and the Spanish Government (Real Decreto 53/2013, de 1 de febrero, por el que se establecen las normas básicas aplicables para la protección de los animales utilizados en experimentación y otros fines científicos, incluyendo la docencia).

### Experimental Food Making

Commercial fish feed (L2 Alterna^®^ Skretting, Burgos, Spain), with a size of 2.2 mm, was used as the control diet. L-phenylalanine and L-tyrosine (dry powder) were purchased from ThermoFisher (Kandel, Germany). The commercial control diet was finely ground and mixed with AA and later water (300 mL Kg^–1^ dry feed). The amount of phenylalanine and tyrosine in each experimental diet was 5% (on dry feed) except for the control diet which did not have any amino acid. The mixture was thread pelleted into 2 mm diameter and 20–25 cm length strips. These were cut to get 2–3 mm size pellets. Finally, these food strips were dried at 30°C for 48 h and stored at 4°C.

Analysis of AA was performed as reported previously (Herrera et al., 2020; Salamanca et al., 2020). According to the above method, the final amino acid concentrations in the experimental diets are shown in Table 1.

**TABLE 1:** Amino acid composition (g amino acid/Kg wet food) for every experimental fish feed.

Amino acid	Control	5% Phe	5% Tyr
Alanine	0.52	0.76	0.76
Glycine	0.98	1.30	1.10
Valine	0.53	0.71	0.72
Leucine	0.63	0.90	0.87
Isoleukin	0.99	1.23	1.21
Proline	2.39	3.46	3.33
Threonine	0.01	0.01	0.01
Aspartic acid	1.14	1.58	1.50
Methionine	0.09	0.12	0.11
Glutamic acid	4.91	6.69	6.37
Phenylalanine	1.05	3.74	1.41
Lysine	0.84	1.24	1.09
Tyrosine	0.89	1.37	5.21

### Plasma Analysis

Plasma glucose, proteins, lactate, and catecholamines (adrenaline, noradrenaline, and dopamine) levels were measured using commercial kits from Química Analitica Aplicada S.A. (QCA Glucose Liquid Ref. 998,225, QCA Total Proteins Ref. 997,180, Tarragona, Spain), Spinreact (Lactate Ref. 1,001,330, Barcelona, Spain), and 2-CAT (A-N) Research ELISA (Ref. BA E-5400, Nordhorn, Germany) adapted to 96-well microplates (Herrera et al., 2012). All assays were performed with a Varioskan Lux reader, using Skantt software for microplate readers v.6.0 (Thermo Fisher Scientific, MA, United States). Plasma cortisol levels were quantified by an ELISA kit (EA65, Oxford Biomedical Research, MI, United States) modified and adapted to fish. Cortisol was extracted from 20 μL plasma in 200 μL diethyl ether. The lower limit of detection (88.2% of binding) was 0.005 ng mL^–1^ plasma. The interassay coefficient of variation was 9.8%, while the mean intraassay coefficient of variation was 4.6%. The mean percentage of recovery was 90%. The main cross reactivities (>5%; given by the supplier) were detected with prednisolone (66.9%), 11-deoxycortisol (58.1%), cortisone (15.9%), prednisone (13.7%), and 17-hydroxyprogesterone (5.4%).

### Tissue Analysis

For brain catecholamines, 20–50 mg of brain tissue was homogenized by ultrasonic disruption with 150 μL of perchloric acid (PCA), and then mixed with 150 μL of potassium dichromate 0.15 M. Following this, the mixture was centrifuged (1200 *g*; 4°C; 10 min) and the supernatant removed. The precipitate was air-dried for 2 h at 25°C. Finally, 250 μL of distilled water was added to the tube and the solution used for ELISA kit determination according to the above section “Plasma Analysis.”

For analyzing Phe and Tyr concentrations, tissue samples (20–50 mg) were dissolved in an acid hydrolysis and basic hydrolysis. The solution was diluted to 1:20 to avoid high concentration. Derivatization procedure was based on the method described by Zhao et al. (2017) with some modifications. An aliquot (100 μL) of working standard solution or sample extract was placed in a 2-mL vial, and 400 μL of a NaOH (3%):ethanol:pyridine (60:32:8) mixture and 40 μL of ethyl chloroformate were added. It was capped and vigorously shaken with a vortex mixer for 60 s at room temperature. Gas evolution (carbon dioxide) usually occurs. Then, 200 μL of chloroform (containing 1% ethyl chloroformate, ECF) and 200 μL 50 mM sodium bicarbonate solution were added. The derivatives were extracted into the organic phase by striking the tube against a pad for about 30 s. The organic phase was dried with anhydrous sodium sulfate. Another 200 μL of chloroform (containing 1% ECF) were added and shaken for 30 s. The organic phase was dried with anhydrous sodium sulfate, and transferred to a new gas chromatography (GC) vial.

Aliquots (1 μL) of the derivative extracts were injected into a Shimadzu GCMS-TQ8030 equipped with an Agilent HP-5MS fused silica capillary column (60 m × 0.25 mm i.d., 0.25 mm film thickness). The gas chromatograph system was equipped with a split/splitless injection port operating in Splitless mode. The column was kept at 120°C for 1 min, ramped at 15°C min^–1^ to 285°C and held for 3 min, then the temperature was increased at 15°C min^–1^ to 300°C and held for 2 min. The carrier gas was helium with a constant flow of 1.2 mL min^–1^. The temperature of the injector, transfer line, and ion source was maintained at 250, 290, and 230°C, respectively, and a solvent delay of 10 min was selected. The mass spectrophotometry (MS) was tuned to *m/z* 69, 219, and 502 for EI corresponding to perfluorotributylamine (PFTBA). Each compound was identified using three characteristic ions, a quantifier, two qualifier ions, and the relative intensity of qualifier to quantifier ion (±20%). For phenylalanine *m/z* 176 (quantifier), *m/z* 102, *m/z* 91, and tyrosine *m/z* 107 (quantifier), *m/z* 192, *m/z* 264 were used. Quantification was conducted by the standard external calibration method following the same procedure for all samples. The detection limits of phenylalanine and tyrosine were 0.35 and 0.05 ng mL^–1^, respectively (Huang et al., 1993; Qiu et al., 2007; Zhao et al., 2017).

### Statistical Analysis

Normality and homoscedasticity of all data sets were checked through the Kolmogorov–Smirnov and Levene tests, respectively (SPSS v.21.0, IBM, Armonk, NY, United States). Body weight and total length values over time were adjusted to a linear equation (best curve fit) by curve estimation regression models (SPSS v.21.0, IBM, Armonk, NY, United States). Differences among curve slopes from weight and length were detected through an ANOVA analysis using treatment (control or supplemented feed) as factor and time as covariate. Differences among treatments (marked fish) were detected through paired-samples T or measures repeated ANOVA tests. Data are expressed as mean ± SEM. The significance level was 0.05.

## Results

### Biometric Parameters

**FIGURE 1:**
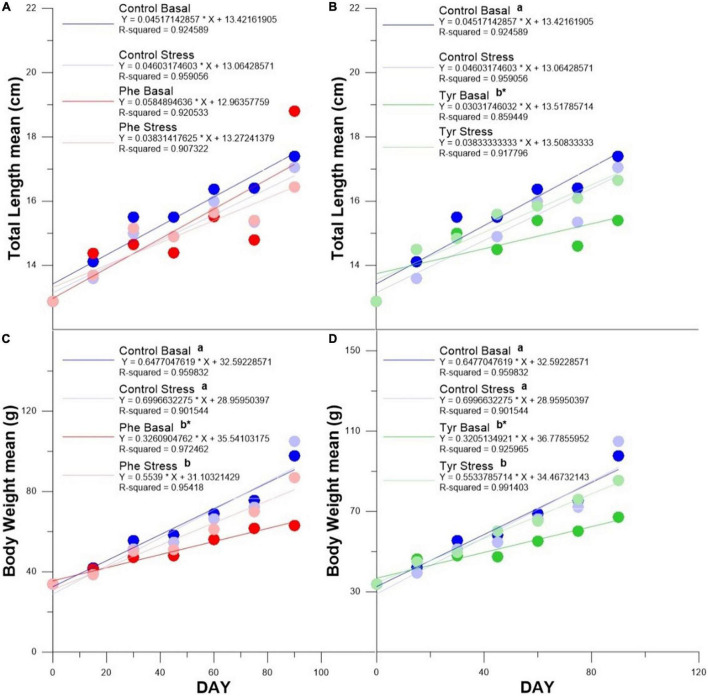
Total length curve for Phe enriched **(A)** and Tyr enriched **(B)** diets. Body weight curve for Phe enriched **(C)** and Tyr enriched **(D)** diets. Asterisks indicate significant differences in curve slope between basal and stress for the same feed, and different letters indicate differences in curve slope between control and phenylalanine or tyrosine within every state (stress or basal).

There were no differences in the slope of the length curve in the specimens fed with Phe compared to the control (Figure 1A). The slope of the length curve in fish fed tyrosine at basal (Tyr basal) was significantly less than that of fish fed a control diet. Furthermore, the slope of the length curve was less than that of the fish subjected to stress and fed the same amino acid (Figure 1B). With respect to weight, the slope of the curve of the specimens fed AA was significantly lower than those fed the control diet both in stress and basal state. Samples fed with AA and subjected to stress showed a significantly greater weight curve than those fed with the same amino acid within the basal state (Figures 1C,D).

The fish fed control diet and in the basal state presented higher condition factor than those fed amino acid-enriched diets (Table 2). Moreover, fish fed control diet and subjected to stress presented a higher condition factor than those fed amino acid-enriched diets. In the basal state, the specimens fed a diet supplemented with phenylalanine showed a higher hepatosomatic index than those fed control diet. The specific growth rate did not present any significant difference between any of the experimental group.

**TABLE 2:** Biometric parameters for the experiment (mean ± SE).

	SGR (% day^–1^)	CF	*K*	HSI
Ctrl basal	0.89 ± 0.47	2.32 ± 2.12	1.89 ± 0.09^a*^	0.94 ± 0.18^a^
Ctrl stress	1.06 ± 0.41	1.15 ± 0.63	2.10 ± 0.02^a^	1.21 ± 0.54
Phe basal	0.55 ± 0.19	1.89 ± 0.40^*^	1.58 ± 0.17^b^	1.24 ± 0.29^b^
Phe stress	0.73 ± 0.38	1.21 ± 0.53	1.94 ± 0.09^b^	1.18 ± 0.51
Tyr basal	0.63 ± 0.20	1.68 ± 0.39	1.85 ± 0.14^a^	1.15 ± 0.33^ab^
Tyr stress	0.82 ± 0.21	1.67 ± 0.55	1.86 ± 0.01^b^	1.10 ± 0.29

*SGR, specific growth rate; CF, conversion factor; K, condition factor; HSI, hepatosomatic index. Different letters indicate significant differences between the different diets. Asterisks indicate significant differences between stress and basal within every diet.*

### Classical Stress Markers Over Time

**FIGURE 2:**
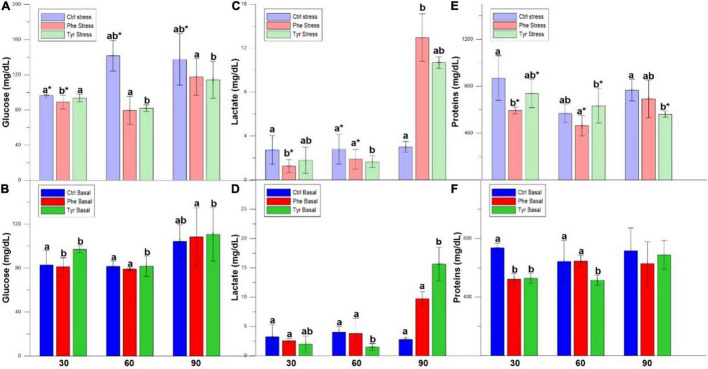
Glucose **(A,B)**, lactate **(C,D)**, and proteins **(E,F)** values (mean ± SEM) for the experimental cultures. Different letters indicate significant differences between the different diets. Asterisks indicate significant differences for the same diet between stress and basal.

After 30 days of feeding, the plasma glucose values were higher in the specimens subjected to stress except for those fed with a diet supplemented with Tyr (Figure 2A). Furthermore, the control was significantly different from those fed the amino-acid-supplemented diet (Figures 2A,B). In the fish subjected to stress and fed the diet enriched with phenylalanine, lactate was significantly lower compared to the rest of the specimens in the basal state (Figures 2C,D). The proteins were significantly higher in the specimens subjected to stress with respect to the basal one, and also in the specimens fed with the control diet with respect to those fed with a diet enriched with AA (Figures 2E,F). After 60 days of feeding, the plasma glucose in the fish fed the diet supplemented with tyrosine was significantly different with respect to the other treatments (Figure 2A). For lactate and protein, the values for those fed phenylalanine and subjected to stress were different from those fed the same diet at basal. After 90 days, the plasma metabolites of the tyrosine-fed samples were significantly different from the other treatments, except for proteins at basal, where there was no difference.

**FIGURE 3:**
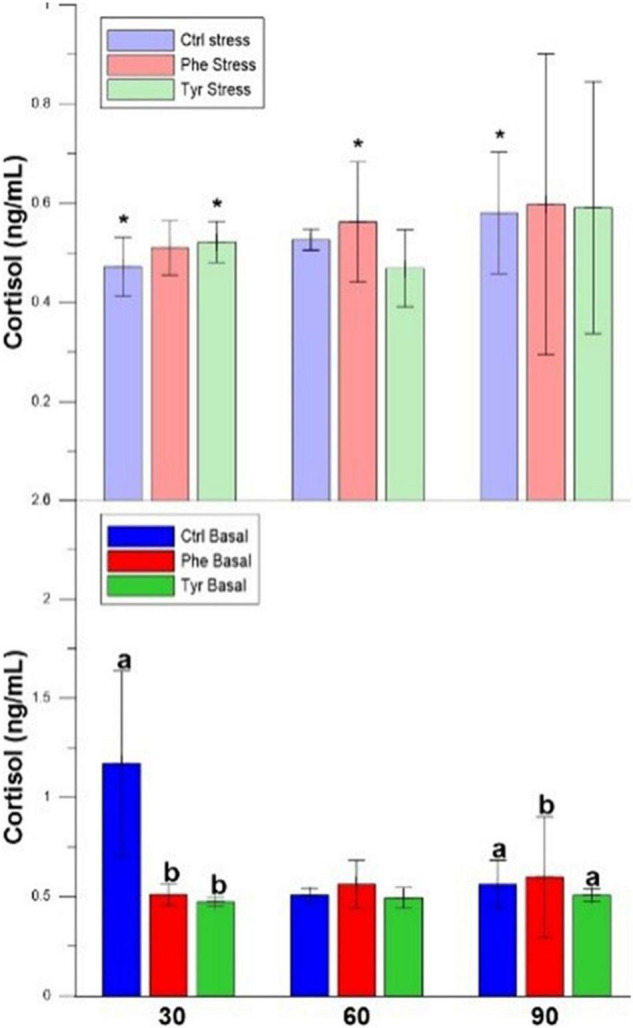
Cortisol values for the experimental cultures (mean ± SEM). Different letters indicate significant differences between the different diets. The asterisks indicate significant differences for the same diet between stress and basal.

The plasma cortisol of those specimens subjected to stress did not show significant differences between the control and those fed the diet supplemented with AA at all sampling points. During days 30 and 90, the cortisol values for stressed fish were significantly different when compared with the basal treatment ones for the control diet. In basal state, plasma cortisol in specimens fed the control diet was higher than those fed with AA on day 30. During day 90, this hormone in specimens fed the diet supplemented with phenylalanine were significantly higher than the other treatments (Figure 3).

### End-Point Hormone Concentrations

**FIGURE 4:**
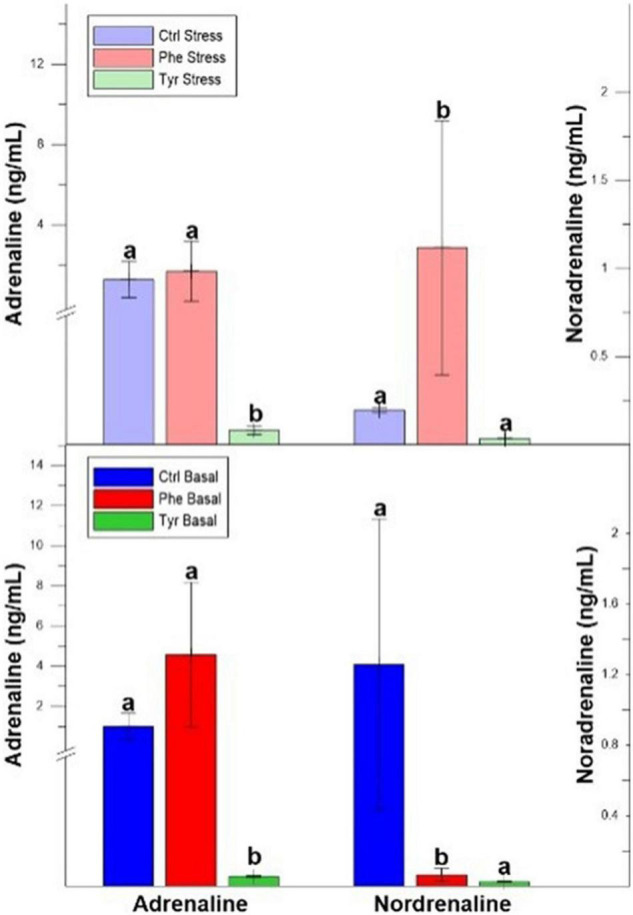
Plasma adrenaline and noradrenaline after 90 days values (mean ± SEM). Different letters indicate significant differences between the different diets.

The plasma adrenaline values after 90 days were significantly lower in the specimens fed the diet supplemented with tyrosine for both states (stress and basal). At the end of the experiment, the plasma noradrenaline values in the specimens fed phenylalanine diet, in both states, were significantly different than the other treatments (Figure 4).

**FIGURE 5:**
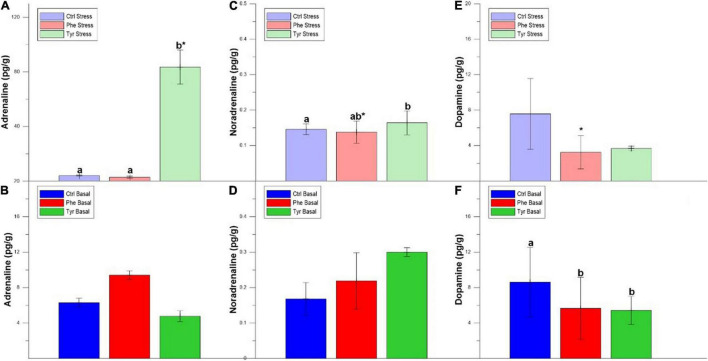
Brain adrenaline **(A,B)**, noradrenaline **(C,D)**, and dopamine **(E,F)** values after 90 days (mean ± SEM), per grams of tissue. Different letters indicate significant differences between the different diets. Asterisks indicate significant differences for the same diet between stress and basal.

Figure 5 shows the catecholamine hormone values in the brain. The adrenaline (Figure 5A) and noradrenaline (Figure 5C) values of the specimens subjected to stress and fed the diet supplemented with tyrosine were significantly higher than those fed the control diets or supplemented with phenylalanine. For noradrenaline (Figures 5C,D) and dopamine (Figures 5E,F), the specimens fed the diet supplemented with phenylalanine showed significant differences between the stress and basal state. In the basal state, the specimens fed the control diet presented dopamine levels that were higher than the other treatments (Figure 5F).

**FIGURE 6:**
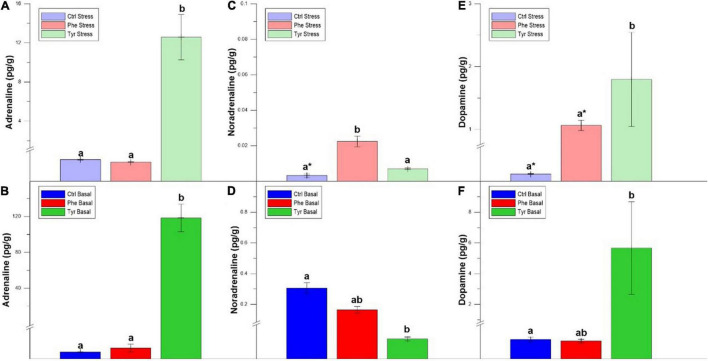
Head kidney adrenaline **(A,B)**, noradrenaline **(C,D)**, and dopamine **(E,F)** values after 90 days (mean ± SEM). Different letters indicate significant differences between the different diets. The asterisks indicate significant differences for the same diet between stress and basal.

There were significant differences in adrenaline for the head kidney both in stress and basal state, with the concentration of this hormone being higher in those fed Tyr (Figures 6A,B). The concentration of noradrenaline in those subjected to stress was higher in those fed with the diet supplemented with Phe compared to the other treatments (Figure 6C). However, in those specimens that were not subjected to stress and fed with Tyr, the concentration of this hormone was inferior to the other treatments (Figure 6D). The dopamine values in the samples fed with tyrosine were significantly higher than in the rest of the diets for both stress and basal states (Figures 6E,F). Furthermore, there were significant differences between stress and basal treatments for fish fed the control diet or the phenylalanine-supplemented diet.

**FIGURE 7:**
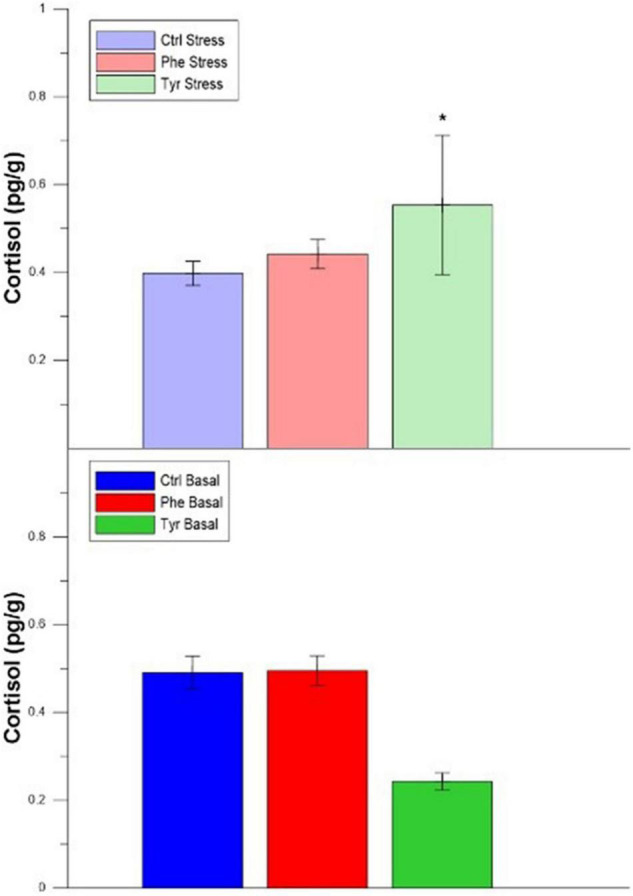
Head kidney cortisol after 90 days (mean ± SEM). The asterisks indicate significant differences for the same diet between stress and basal.

Regarding the cortisol concentration in the head kidney, differences were only detected between stress and basal treatments for fish fed the diet supplemented with tyrosine (Figure 7).

### Phenylalanine and Tyrosine Concentration in Tissues

**FIGURE 8:**
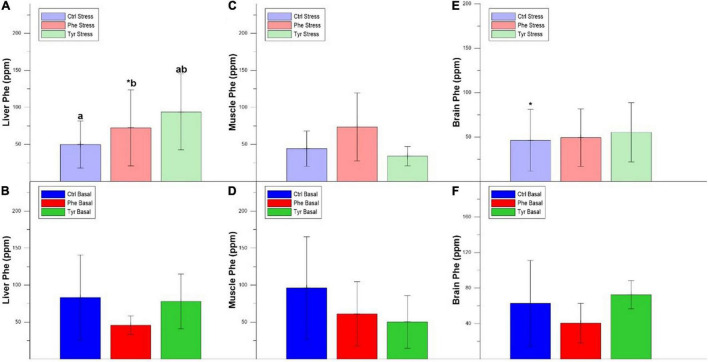
Liver Phe stress **(A)**, liver Phe basal **(B)**, muscle Phe stress **(C)**, and muscle Phe basal **(D)**, brain Phe stress **(E)**, and brain Phe basal **(F)** values after 90 days (mean ± SEM). Different letters indicate significant differences between the different diets. The asterisks indicate significant differences for the same diet between stress and basal.

**FIGURE 9:**
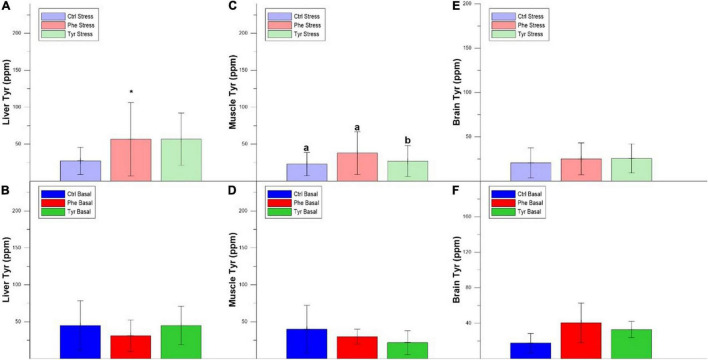
Liver Tyr stress **(A)**, liver Tyr basal **(B)**, muscle Tyr stress **(C)** and muscle Tyr basal **(D)**, brain Tyr stress **(E)**, and brain Tyr basal **(F)** values after 90 days (mean ± SEM). Different letters indicate significant differences between the different diets. The asterisk indicates significant differences for the same diet between stress and basal.

The concentration of Phe in the liver was higher in fish fed the control diet compared to those fed the diet enriched with Phe. In addition, there was a decrease in the liver Phe concentration of the fish subjected to stress compared to the basal within the Phe-supplemented diet (Figures 8A,B). The same happened for the liver Tyr concentration in specimens fed the diet enriched with Phe (Figures 9A,B). Figures 8C,D show the Phe concentration in the muscle, where there was no difference except for fish fed the control diet and where the specimens subjected to stress had a higher Phe concentration. There was no difference in muscle Tyr concentration within any treatment (Figures 9C,D). There was no difference in the brain Phe concentration (Figures 8E,F). However, there were differences in the brain Tyr concentration for the stressed fish fed the control or Phe-enriched diets compared to those fed the Tyr diet (Figures 9E,F).

## Discussion

In our study, Phe and Tyr supplementation in the diet reduced fish growth and biometric parameters, in addition to modulated classical stress markers. It also produced an accumulation of both AA in liver, brain, and muscle that could be limiting to activate some metabolic pathways, since it has been shown that an increase in dietary Tyr decreases the body’s Phe requirements (Li et al., 2009).

The effects of dietary supplementation with Tyr and its metabolic products on the stress system and growth has been studied by various authors (Garg, 2007; Herrera et al., 2017; Salamanca et al., 2021). Phe supplementation in the diet has been studied in a few fish species (*Catla catla, Megalobrama ambycephala*, and *Gadus morhua*) (Zehra and Khan, 2014; Ren et al., 2015; Herrera et al., 2016).

According to Garg (2007), an increase in T4 (thyroxine), a product of Tyr metabolism, causes a decrease in body growth parameters. Similarly, Saavedra et al. (2010) showed that an increase in Phe and Tyr supplementation in the diet of white seabream (*D. sargus*) larvae reduced the growth rate. Something similar happened in our study since the specimens fed the Tyr diet showed a decrease in specific growth rate and growth curve slope. The SGR presented a decreasing tendency in non-stressed fish in the Phe-enriched treatment, which may be due to the Tyr formation pathway that was stimulated from Phe (Li et al., 2009). This is supported by other results (see below) since the accumulation of Tyr in the liver in those specimens was higher than the specimens subjected to stress. Moreover, the continuous feeding through an unbalanced diet (amino acid excess) could result in malnutrition and, hence, poor growth (Wu, 2009).

According to Herrera et al. (2016), the concentration of plasma stress markers (glucose, lactate, and proteins) in Atlantic cod (*G. morhua*) fed diets supplemented with Phe high levels presented a significant drop. Our results showed similar patterns since there was a decrease in glucose, lactate, and proteins with respect to the control at all sampling points. Nevertheless, this did not occur in lactate values after 90 days when there was a significant increase in those specimens fed the diet enriched with AA. According to Li et al. (2009), an increase in the amount of Tyr supplied in the body produces a decrease in the Phe requirement, which causes its accumulation. Consequently, our fish could have an excess of Phe that caused the lactate rise, as stated by Schuck et al. (2015) in humans and mice having phenylketonuria (an increased Phe concentration).

Plasma cortisol only showed differences in non-stressed specimens at 30 and 90 days between the control and the specimens fed amino acid diets and between the specimens fed with Phe and with other treatments, respectively. It is probable that enriched foods could attenuate the cortisol increase related to the stress response since it only varied significantly between basal and stress states in the Phe treatment; in this sense, previous studies have demonstrated that the cortisol levels did not show differences in seabreams (*S. aurata*) which were fed with Phe and Tyr supplemented diets (Salamanca et al., 2021). As the present experiment was based on chronic stress, the absence of cortisol changes after 3 months could have been expectable due to the acclimation of the hypothalamic–pituitary–interrenal (HPI) axis (Montero et al., 1999; Procarione et al., 1999; Haukenes and Barton, 2004; Barton et al., 2005). However, the differences between stressed and non-stressed control fish were evident at the end of the experimental culture, as well as the attenuating effects of the enriched diets.

It has been shown that the supplementation of AA in the diet attenuates stress in *A. regius* and *M. ambycephala* (Ren et al., 2015; Fernández-Alacid et al., 2019). In the present work, this effect produced by Phe and Tyr may be because both the AA are precursors of hormones involved in stress processes, such as adrenaline, noradrenaline, and dopamine (Zehra and Khan, 2014). However, Salamanca et al. (2021) did not detect significant differences in plasma adrenaline and noradrenaline in seabreams fed amino acid supplements. These differences may be because the fish were fed for a short period of time (7 days) in that work, as opposed to the 90 days of feeding in our experiment.

The stress response involves the recognition of a threat by the central nervous system, with a rapid increase in plasma catecholamines (Reid et al., 1998; Gallo and Civinini, 2003). In turn, the activation of these endocrine pathways is derived from changes in the plasma and tissue metabolites to cope with the energy imposed by the stressor. In teleost fish, the activity of the HPI axis, involved in the stress response, is stimulated by noradrenaline (Höglund et al., 2000; Li et al., 2009).

In our stress condition, plasma adrenaline and noradrenaline values were higher in those specimens fed the Phe-supplemented diet. It has been reported that there is an inverse relationship between Phe concentration and norepinephrine and dopamine values (Güttler and Lou, 1986). In fact, the increase of Phe concentration in the diet decreased the concentration of catecholamine hormones in plasma, since increasing the concentration of this amino acid in the blood favors its passage through the blood–brain barrier compared to other AA, including tyrosine, hence, the synthesis of catecholamines is not stimulated (Salamanca et al., 2021). However, this did not occur in the present study. This may be due to the fact that, when feeding for a long period with Phe-enriched diets, this amino acid is catabolized to Tyr, which causes the catecholamine hormone formation pathway to be stimulated. Furthermore, in our work, this favored the accumulation of Tyr in the different tissues. The brain adrenaline and noradrenaline concentrations were slightly higher in those specimens fed Tyr-enriched diets. In this sense, the accumulation of Tyr in brain could have enhanced the catecholamine formation (Damasceno-Oliveira et al., 2007).

An increase in adrenaline and dopamine concentrations occurred in the head kidney, which also could be due to the increase in the concentration of Tyr in this tissue (Damasceno-Oliveira et al., 2007). The level of dopamine present in the head kidney of seabream was higher in the diet supplemented with Tyr; hence this amino acid enhanced this hormonal response, since Tyr seems to be limiting for the production of the catecholamine hormones (Cotoia et al., 2014). According to Øverli et al. (1999) and Höglund et al. (2001), an increase in L-Dopa (precursor of dopamine) enhanced the plasma cortisol in Arctic char (*Salvelinus alpinus*). In this sense, in our study there was a cortisol increase in the head kidney of fish fed the diet supplemented with Tyr and subjected to stress, coinciding with a dopamine raise in this tissue.

## Conclusion

In conclusion, to our knowledge, this is the first work on the effects of the Phe- or Tyr-enriched diets on the response to chronic stress in fish. Both the AA altered the stress response and the zootechnical parameters in the seabream (*S*. *aurata*). The hormonal stress markers were significantly attenuated in different tissues. Nevertheless, the experimental diets could decrease the growth parameters due to the unbalanced formulation (amino acid excess) and the long feeding time. Therefore, it is highly probable that this type of feed supplements should be applied in short-time basis, just before a stressing condition, as previously assayed in previous works. Future research should be focused on finding a balanced diet, including nutritional supplements for long-term feeding which does not affect growth and, in addition, improve the chronic stress response. In addition, this research line could study the inclusion of both AA for detecting synergistic or antagonistic effect.

## Data Availability Statement

The original contributions presented in the study are included in the article/supplementary material, further inquiries can be directed to the corresponding author.

## Ethics Statement

The animal study was reviewed and approved by REGA Code ES210210000303.

## Author Contributions

NS and MH: methodology. IG and EM: software and formal analysis. IR: resources. NS: writing – original draft preparation. NS, OM, IG, EM, IR, and MH: writing – review and editing. MH: supervision and project administration. MH and OM: funding acquisition. All authors have read and agreed to the published version of the manuscript.

## Conflict of Interest

The authors declare that the research was conducted in the absence of any commercial or financial relationships that could be construed as a potential conflict of interest.

## Publisher’s Note

All claims expressed in this article are solely those of the authors and do not necessarily represent those of their affiliated organizations, or those of the publisher, the editors and the reviewers. Any product that may be evaluated in this article, or claim that may be made by its manufacturer, is not guaranteed or endorsed by the publisher.
